# Coculture of endothelial progenitor cells and mesenchymal stem cells enhanced their proliferation and angiogenesis through PDGF and Notch signaling

**DOI:** 10.1002/2211-5463.12317

**Published:** 2017-10-16

**Authors:** Tangzhao Liang, Lei Zhu, Wenling Gao, Ming Gong, Jianhua Ren, Hui Yao, Kun Wang, Dehai Shi

**Affiliations:** ^1^ Department of Orthopaedic Surgery the Third Affiliated Hospital of Sun Yat‐sen University Guangzhou China; ^2^ Department of Plastic and Reconstructive Surgery the Third Affiliated Hospital of Sun Yat‐sen University Guangzhou China; ^3^ Department of Periodontology Faculty of Dentistry Prince Philip Dental Hospital The University of Hong Kong China; ^4^ Department of Orthopedic Surgery Shenzhen Hospital of Southern Medical University China

**Keywords:** coculture, endothelial progenitor cell, enhanced regeneration, mesenchymal stem cell, platelet derived growth factor, signaling pathway

## Abstract

The beneficial effects of combined use of mesenchymal stem cells (MSCs) and endothelial progenitor cells (EPCs) on tissue repair and regeneration after injury have been demonstrated, but the underlying mechanism remains incompletely understood. This study aimed to investigate the effects of direct contact coculture of human bone marrow‐derived EPCs (hEPCs)/human bone marrow‐derived MSCs (hMSCs) on their proliferation and angiogenic capacities and the underlying mechanism. hEPCs and hMSCs were cocultured in a 2D mixed monolayer or a 3D transwell membrane cell‐to‐cell coculture system. Cell proliferation was determined by Cell Counting Kit‐8. Angiogenic capacity was evaluated by *in vitro* angiogenesis assay. Platelet‐derived growth factor‐BB (PDGF‐BB), PDGF receptor neutralizing antibody (AB‐PDGFR), and DAPT (a γ‐secretase inhibitor) were used to investigate PDGF and Notch signaling. Cell proliferation was significantly enhanced by hEPCs/hMSCs 3D‐coculture and PDGF‐BB treatment, but inhibited by AB‐PDGFR. Expression of cyclin D1, PDGFR, Notch1, and Hes1 was markedly enhanced by PDGF‐BB but inhibited by DAPT. *In vitro* angiogenesis assay showed that hEPCs/hMSCs coculture and PDGF‐BB significantly enhanced angiogenic capacity, whereas AB‐PDGFR significantly reduced the angiogenic capacity. PDGF‐BB increased the expression of kinase insert domain receptor (KDR, an endothelial marker) and activated Notch1 signaling in cocultured cells, while DAPT attenuated the promoting effect of PDGF‐BB on KDR expression of hEPCs/hMSCs coculture. hEPCs/hMSCs coculture enhanced their proliferation and angiogenic capacities. PDGF and Notch signaling pathways participated in the promoting effects of hEPCs/hMSCs coculture, and there was crosstalk between these two signaling pathways. Our findings should aid understanding of the mechanism of beneficial effects of hEPCs/hMSCs coculture.

**Abbreviations**
2D: two‐dimensional
3D: three‐dimensional
AM: adipogenic medium
ECs: endothelial cells
EPCs: endothelial progenitor cells
FACS: fluorescence‐activated cell sorting
FITC: fluorescein isothiocyanate
HBMECs: human brain microvascular endothelial cells
hEPCs: human bone marrow‐derived EPCs
HLA‐DR: human leukocyte antigen
hMSCs: human bone marrow‐derived MSCs
HUVECs: human umbilical vein endothelial cells
MSCs: mesenchymal stem cells
N1ICD: Notch1 intracellular domain

During tissue repair, adequate angiogenesis is a crucial precondition for success in repair and functional recovery of injured tissue due to the requirement for nutrients and oxygen from blood supply 1, 2. Currently, cell‐based therapeutic strategies have been widely applied in tissue repair and regeneration after injury 3. Endothelial progenitor cells (EPCs) are a group of circulating cells derived from bone marrow, adult peripheral blood, and umbilical cord blood, which can differentiate into mature endothelial cells (ECs), and play important roles in angiogenesis, neovascularization, and vascular endothelial repair 4. Mesenchymal stem cells (MSCs) are multipotent stem cells capable of self‐renew, differentiating, and participating in angiogenesis. Both MSCs and EPCs take part in vascularization and tissue repair and have been widely employed for cell‐based therapy in both preclinical studies 5, 6 and clinical trials 7, 8.

Even though many previous studies utilized a single type of cells for transplantation, however, some studies have suggested that coculture or transplantation of more than one type of cells may improve the biological properties of stem cells 9, 10. It has been demonstrated that coculture of human umbilical vein endothelial cells and MSCs can form a vascular tissue‐like network *in vitro* through the induction of VEGF production 11. When MSCs were cocultured with ECs, the secreted TGF‐β stimulated their differentiation into pericytes and smooth muscle cells, both of which were involved in blood vessel formation 12. Furthermore, cell‐to‐cell interaction promotes rat MSC differentiation into EC via activation of TACE/TNF‐alpha signaling 13. These findings indicated that cell–cell coculture may activate multiple signaling pathways to enhance the biological effects of MSCs. To improve the therapeutic efficacy, therefore, several studies involving a combination of EPCs and MSCs for coculture or cotransplantation have been reported. The purpose of the combined use strategy is to achieve synergistic effects on angiogenesis and tissue regeneration. It has been revealed that direct cell‐to‐cell contact in MSC‐based coculture significantly enhanced the biological properties of MSCs 14. Fu *et al*. 15 have demonstrated that coculture of peripheral blood‐derived MSCs and EPCs on strontium‐doped calcium polyphosphate scaffolds can enhance osteogenic and angiogenic markers and generate a vascularized engineered bone. Likewise, Li and Wang 16 have reported that canine bone marrow‐derived MSCs and EPCs cocultured in a direct contact coculture system promote osteogenesis and angiogenesis. Very recently, Sun *et al*. 17 conducted a meta‐analysis including five controlled preclinical studies on cotransplantation in animal models of disease. Their results showed that compared with MSC‐alone group, cotransplantation of EPCs and MSCs significantly enhanced angiogenesis, bone regeneration, vessel revascularization, and tissue repair in cerebrovascular disease model 17. However, although the beneficial effects of combined use of MSCs and EPCs have been demonstrated, the underlying molecular mechanism is still not fully understood.

PDGF and Notch signaling pathways have been shown to be involved in the pre‐ and postnatal vasculogenesis and/or angiogenesis 18, 19. PDGF signaling is critical for vascular development and blood vessel homeostasis 20. It has been shown that PDGFs are potent mitogens for mesenchymal cells and are involved in angiogenic induction 21. PDGF signaling is important for differentiation and growth of MSCs 22. PDGF isoforms exert their biological effects through the activation of two tyrosine kinase receptors, PDGFR‐α and PDGFR‐β, which are expressed on MSCs and EPCs 23. Meanwhile, in addition to improving angiogenic functions, exogenous PDGF‐BB also strongly induced the proliferation and migration of MSCs 24. On the other hand, Notch signaling is a highly conserved, cell–cell signaling pathway involved in cell differentiation 25. Binding with Notch ligands such as Delta and Jagged‐1 induces a proteolytic cleavage of the Notch receptors by the γ‐secretase, ultimately leading to transactivation of the promoters of target genes, such a HES and HEY families 26, 27. Notch signaling also mediates intercellular signals that affect proliferation, survival, and differentiation of ECs 28. In angiogenesis, Notch signaling is essential for vascular development and is involved in the determination of arteriovenous vessel fate, whereas alterations in Notch signaling lead to abnormal vascular development at multiple stages 29. Taken together, all these findings suggested that both PDGF and Notch signaling pathways play important roles in the proliferation and angiogenesis of stem cells.

Nevertheless, it remains to be investigated whether PDGF and Notch signaling pathways participate in the biological functions of cocultured MSCs and EPCs. To elucidate the molecular mechanism underlying the promoting effect of combined transplantation of EPCs and MSCs on angiogenic capacity, this study aimed to investigate the effects of human bone marrow‐derived EPCs (hEPCs)/human bone marrow‐derived MSCs (hMSCs) coculture on their proliferation and angiogenic capacities *in vitro* and whether PDGF and Notch signaling pathways play a role in the biological effects of EPCs and MSCs coculture. Utilizing hEPCs and hMSCs in a two‐dimensional (2D) monolayer mixed and 3D transwell membrane cell‐to‐cell coculture systems, the above issue was investigated.

## Results

### Characterization of isolated hEPCs and hMSCs

**Figure 1:**
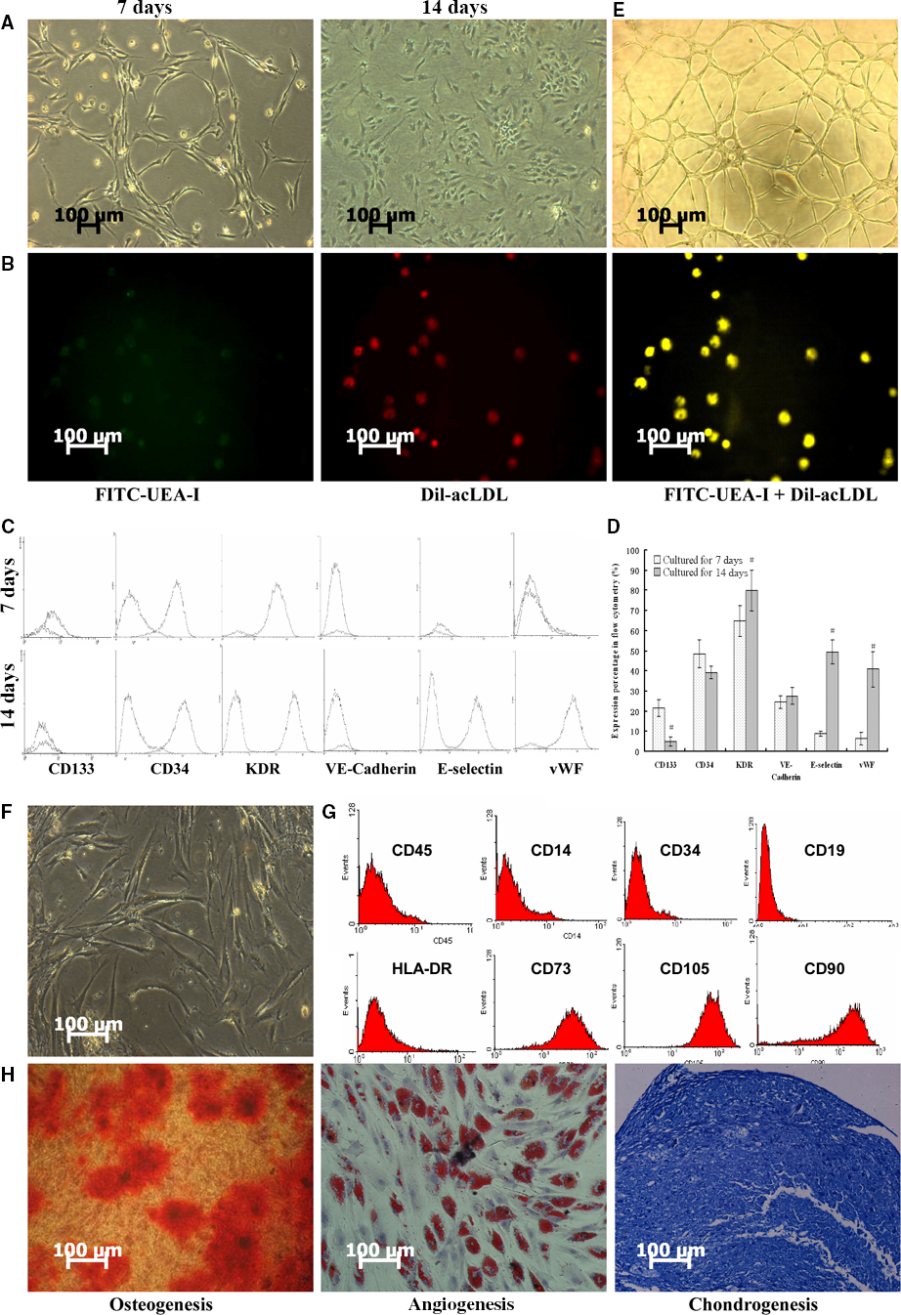
Characterization of isolated hEPCs and hMSCs. (A) Microscopic images of hEPCs on Day 7 and Day 14; (B) hEPCs were double‐stained with Dil‐acLDL and FITC‐UEA‐I and observed by a fluorescence microscopy. Images showed Dil‐acLDL and FITC‐UEA‐I‐labeled hEPCs; (C) FACS analysis for markers of CD133, CD34, KDR, VE‐cadherin, E‐selectin, and vWF expressions on hEPCs on Day 7 and Day 14; (D) quantification of FACS data of hEPCs on Day 7 and Day 14, *n* = 6 for each group, #*P* < 0.05; (E) HEPCs exhibited angiogenic tubule‐like formation on Day 14; (F) microscopic images of passage 3 hMSCs; (G) FACS analysis for markers of CD105, CD73, CD90, CD45, CD14, CD19, CD34, and HLA‐DR on hMSCs; (H) osteogenic, adipogenic, and chondrogenic differentiation assays for hMSCs on Day 21. hMSCs were stained with ARS, Oil red O, and toluidine blue for calcium deposits, lipid droplets, and proteoglycan, respectively.

hEPCs and hMSCs were isolated from human bone marrow samples. The isolated hEPCs exhibited vascular‐like cells on Day 7, changing toward a spindle‐shaped endothelium‐like morphology and assembling in clusters with cobblestone‐like arrangement on Day 14 (Fig. 1A). hEPCs were positive for Dil‐acLDL and FITC‐UEA‐I staining with a double positive rate of 92.7 ± 6.0% (Fig. 1B). FACS analysis showed that hEPCs were positive for mesenchymal markers CD133 and CD34 as well as endothelial markers KDR, VE‐cadherin, E‐selectin, and vWF (Fig. 1C). In addition, the positive cells for KDR, E‐selectin, and vWF significantly increased on Day 14 as compared to Day 7, suggesting a more mature EC phenotype (Fig. 1D, all *P* < 0.05). hEPCs possessed active angiogenic potential in tubule synthesis on Day 14 (Fig. 1E).

Passage 3 hMSCs exhibited typical fibroblastic morphology of hMSCs (Fig. 1F). As shown in Fig. 1G, hMSCs were positive for mesenchymal markers of CD105, CD73, and CD90, but negative for hematopoietic markers CD45, CD14, CD19, and CD34, and human leukocyte antigen (HLA‐DR). Furthermore, the osteogenic, adipogenic, and chondrogenic differentiation assay on Day 21 showed that the cultured hMSCs were of multilineage differentiation potential (Fig. 1G). These results suggested that the cultured hMSC possessed characteristics of mesenchymal stem cells.

### hEPCs/hMSCs coculture and PDGF‐BB enhance proliferation

**Figure 2:**
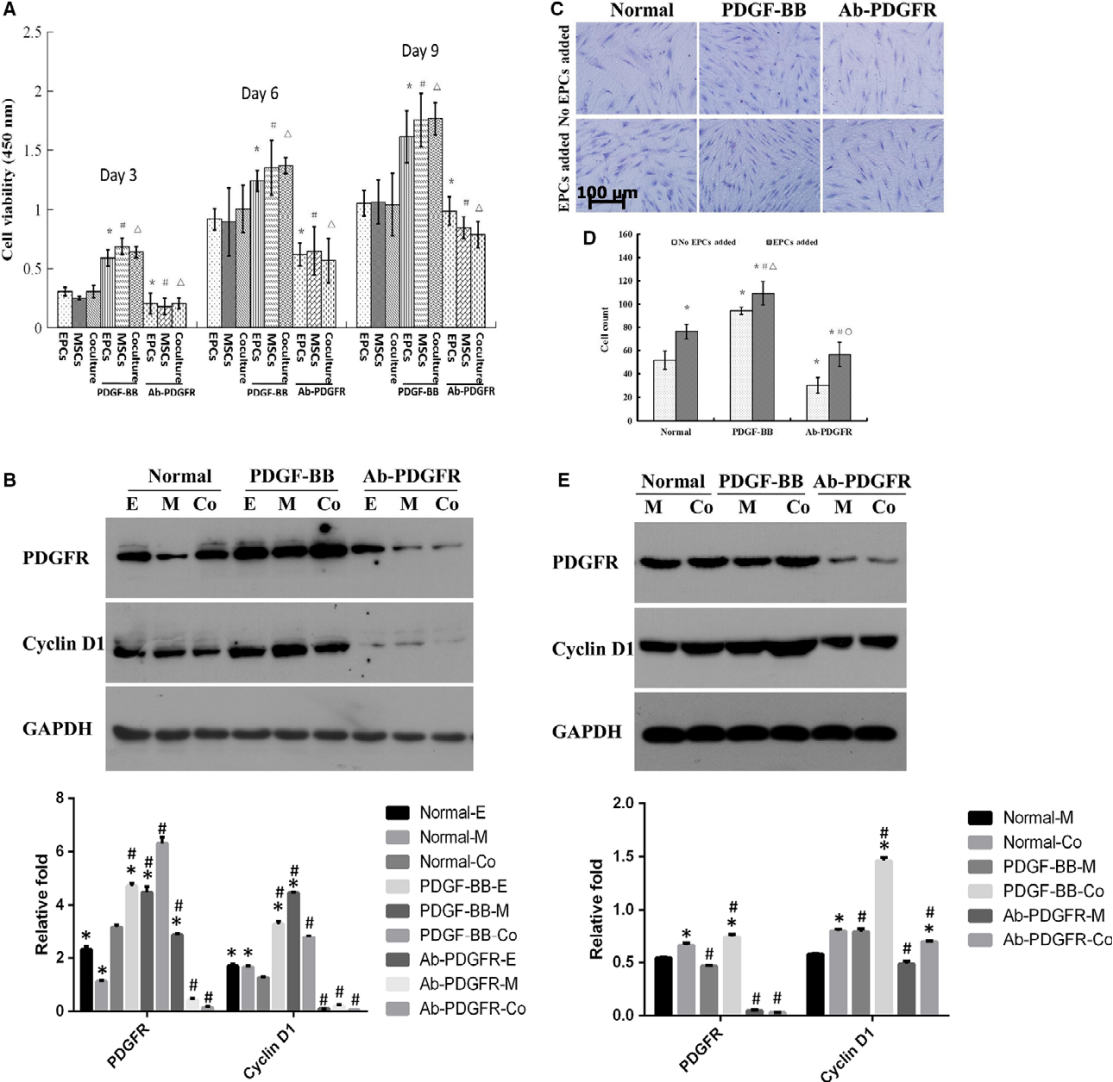
hEPCs coculture and PDGF‐BB enhanced proliferation of hMSCs. (A) Cell viability was determined in a 2D mixed monolayer coculture on Days 3, 6, and 9 as described in Materials and methods. *n* = 6 for each group, *P < 0.05, compared to hEPCs alone; ^#^ *P* < 0.05, compared to hMSCs alone; ^▵^ *P* < 0.05, compared to hEPCs+hMSCs; (B) protein expression levels of cyclin D1 and PDGFR in all groups on Day 6 were determined by western blot. *P < 0.05, compared to coculture group, ^#^ *P* < 0.05, compared to corresponding untreated control; (C) the proliferation of hMSCs on Day 6 in a 3D cell‐to‐cell coculture system, where the hEPCs were cultured on the opposite side of transwell membrane; (D) cell numbers were counted in six random fields (magnification 200×); *P < 0.05, compared to hEPCs alone; ^#^ *P* < 0.05, compared to hEPCs+hMSCs; ^▵^ *P* < 0.05, compared to hEPCs+PDGF‐BB; ^○^ *P* < 0.05, compared to hEPCs+AB‐PDGFR; (E) protein expression level of cyclin D1 and PDGFR in all groups on Day 6 was determined by western blot. *P < 0.05, compared to hMSCs alone, ^#^ *P* < 0.05, compared to corresponding untreated control.

To investigate the effects of hEPCs/hMSCs coculture on the proliferation, a 2D (directly mixed monolayer) coculture system was employed. As shown in Fig. 2A, compared to hEPCs or hMSCs alone, there was no significant effect on cell proliferation of cocultured cells on Days 3, 6, and 9 (all *P* > 0.05). PDGF‐BB treatment significantly enhanced proliferation in single‐cell culture and coculture group, whereas neutralizing antibody against PDGF receptor β (AB‐PDGFR) significantly inhibited proliferation of three groups of cells (all *P* < 0.05). Western blot showed that coculture decreased the protein expressions of cyclin D1 as compared with two single‐cell culture groups (both *P* < 0.05). Meanwhile, the protein levels of cyclin D1 and PDGFR in three groups were consistently upregulated and downregulated by PDGF‐BB and AB‐PDGFR, respectively (all *P* < 0.05, Fig. 2B). These observations indicated that PDGF signaling was implicated in the proliferation of hEPCs and hMSCs.

To eliminate the monolayer coculture‐induced contact inhibition in 2D coculture system, a 3D cell‐to‐cell coculture system was used. As shown in Fig. 2C,D, hEPCs coculture on the opposite side of transwell membrane significantly promoted hMSC proliferation on Day 6, compared to hMSCs cultured alone (*P* < 0.05). The proliferation trends of PDGF‐BB‐ and AB‐PDGFR‐treated cells were consistent with those in 2D coculture. As shown in Fig. 2E, western blot showed that 3D coculture significantly increased cyclin D1 level (*P* < 0.05). PDGF‐BB significantly promoted cyclin D1 and PDGFR expression in both hMSC‐alone and coculture groups (except for PDGFR in hMSC‐only group), whereas AB‐PDGFR significantly inhibited the expressions of these two proteins (compared to untreated counterparts, all *P* < 0.05, Fig. 2E).

### PDGF and Notch signaling pathways were involved in the effect of hEPCs/hMSCs coculture on cell proliferation

**Figure 3:**
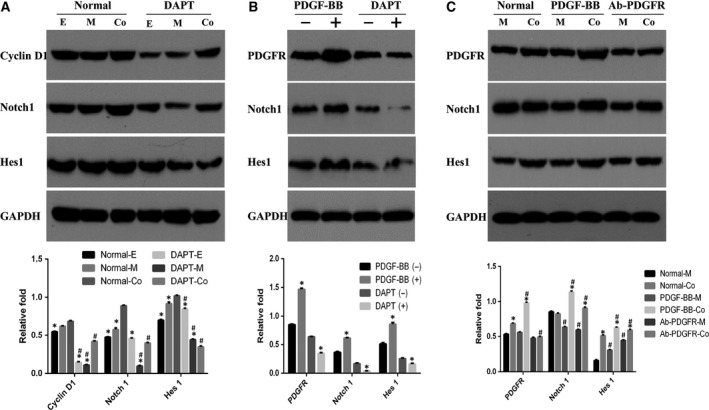
Notch and PDGF signaling participated in the proliferative effects of hEPCs/hMSCs coculture. (A) In the 2D coculture system, the protein expressions Notch1, Hes1, and cyclin D1 of cells with or without DAPT treatment were determined by western blot. *P < 0.05, compared to coculture group, ^#^ *P* < 0.05, compared to corresponding untreated control. (B) Protein levels of PDGFR, Notch1, and Hes1 in hEPCs/hMSCs coculture on Day 6 in the presence of PDGF‐BB or DAPT. *P < 0.05, compared to untreated control. (C) In the 3D coculture, the protein levels of PDGFR, Notch1, and Hes1 in hMSC‐alone or coculture groups on Day 6 in the presence of PDGF‐BB or AB‐PDGFR were determined by western blot. *P < 0.05, compared to hMSCs group, ^#^ *P* < 0.05, compared to corresponding untreated control.

Next, we determined whether Notch signaling pathway plays a role in the molecular mechanism underlying the proliferation‐promoting effect of hEPCs/hMSCs coculture. As shown in Figs 3A and 2D coculture significantly increased the expression of Notch1 as compared with the single type of cell culture groups (both *P* < 0.05), indicating that Notch signaling pathway was implicated in direct contact culture. To further confirm the involvement of Notch signal in the proliferation of hEPCs/hMSCs coculture, a γ‐secretase inhibitor, DAPT, was used to block Notch signaling. DAPT significantly decreased the expressions of cyclin D1, Notch1, and Hes1 (a transcriptional target of Notch signaling) in hMSC‐only group and the coculture groups (Fig. 3A, all *P* < 0.05).

As our results also revealed that PDGF signaling was implicated in the proliferation of hEPCs and hMSCs, we further investigated whether there was a relationship between PDGF and Notch signaling pathways in cocultured hEPCs and hMSCs. The results showed that in addition to protein level of PDGFR, PDGF‐BB significantly enhanced the expressions of Notch1 and Hes1 (all *P* < 0.05, Fig. 3B). Likewise, when Notch signaling was inhibited with DAPT, expression levels of PDGFR, Notch1, and Hes1 declined coincidently (all *P* < 0.05, Fig. 3B). These findings suggested that there was a relationship between PDGF and Notch signaling pathways in cocultured cells. In the 3D coculture system, hEPCs/hMSCs coculture significantly increased the expressions of PDGFR and Hes1 on Day 6 (both *P* < 0.05, Fig. 3C). In addition, PDGF‐BB further elevated the levels of Notch1, Hes1, and PDGFR in cocultured cells (all *P* < 0.05, Fig. 3C). When PDGFR was blocked, the expression of PDGFR showed no significant differences between hMSC‐alone and cocultured groups (*P* > 0.05, Fig. 3C). These observations suggest that the activation of PDGF and Notch signaling pathways was associated in 3D coculture cells.

### hEPCs/hMSCs coculture and PDGF‐BB enhanced angiogenic capacity

**Figure 4:**
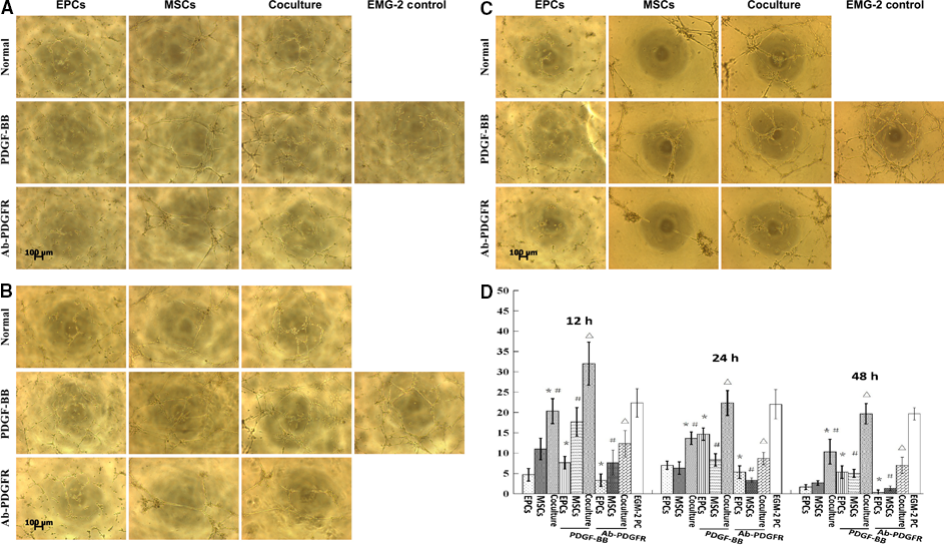
hEPCs coculture and PDGF‐BB enhanced angiogenic capacity of hMSCs. *In vitro* angiogenesis was performed in hEPCs, hMSCs, and cocultured cells with or without PDGF‐BB or Ab‐PDGFR at 12 (A), 24 (B), and 48 (C) hours. hMSCs treated with endothelial cell growth medium (EGM‐2) were used as a positive control (EGM‐2‐PC). (D) The average number of tubules was determined in five independent fields at 100× magnification for each well. *P < 0.05, compared to hEPC‐alone group; ^#^P < 0.05, compared to hMSC‐alone group; ^▵^ *P* < 0.05, compared to coculture group.

To determine the effect of hEPCs/hMSCs coculture on their angiogenic capacity, *in vitro* angiogenesis assay was used. The data of angiogenesis assay at 12, 24, and 48 h are shown in Fig. 4A, 4B, and 4C, respectively, and the tubules quantification data are shown in Fig. 4D. Compared with hEPCs or hMSCs alone, hEPCs/hMSCs coculture significantly improved capillary‐like formation at all the three time points (all *P* < 0.05, Fig. 4D). Images demonstrated a significant increase in tubule cross‐sectional diameter and junction area in coculture group as compared with either hEPCs or hMSC‐alone group at three time points (Fig. 4A–C). In addition, PDGF‐BB treatment significantly improved the amount and diameter of tubules in all groups, whereas PDGFR‐β antibody significantly reduced the angiogenic capacity in all groups as compared with their untreated counterparts (all *P* < 0.05, Fig. 4D). These results suggested that hEPCs/hMSCs coculture and PDGF‐BB enhanced angiogenic capacity.

### PDGF and Notch signaling pathways were involved in angiogenesis of cocultured cells

**Figure 5:**
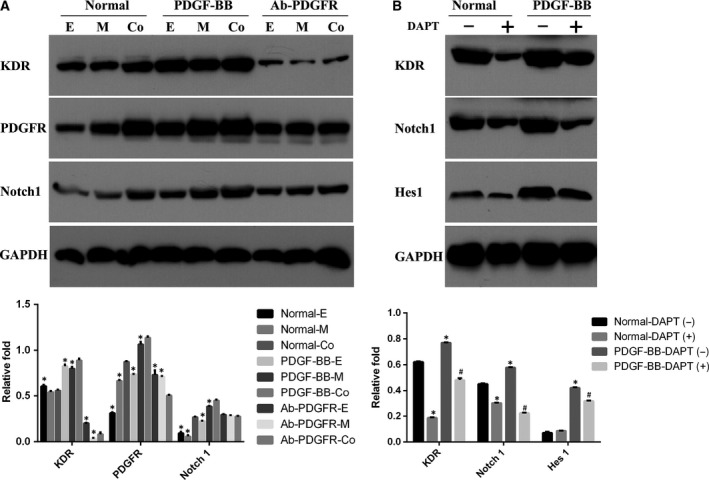
PDGF and Notch signaling pathways were involved in angiogenesis of cocultured cells. (A) Protein levels of KDR, Notch1, and PDGFR in endothelial differentiation of hEPCs, hMSCs, and cocultured cells with or without PDGF‐BB or Ab‐PDGFR on Day 3. *P < 0.05, compared to coculture group. (B) Protein levels of KDR, Notch1, and Hes1 in hEPCs/hMSCs coculture with or without PDGF‐BB and DAPT inhibitor on Day 3. *P < 0.05, compared to normal‐DAPT(‐) group. ^#^ *P* < 0.05, compared to PDGF‐BB‐DAPT(‐) group.

As PDGF‐BB enhanced angiogenic capacity, we further investigated whether Notch signaling pathways were also involved in the angiogenic capacity of cocultured cells. As shown in Fig. 5A, hEPCs/hMSCs coculture and PDGF‐BB markedly increased the levels of KDR (an endothelial marker) and PDGFR (except for KDR in coculture group, all *P* < 0.05). Compared to hMSCs or hEPCs alone, hEPCs/hMSCs coculture markedly increased expression of Notch1 protein (*P* < 0.05, Fig. 5A), suggesting that PDGF and Notch signaling pathways were involved in angiogenesis of hEPCs/hMSCs coculture. We further investigated the relationship between PDGF and Notch signaling pathways. When PDGF signaling was activated by PDGF‐BB, the KDR level elevated in cocultured cells, along with increased activation of Notch1/Hes1 signaling (all *P* < 0.05 compared with untreated control, Fig. 5B). Meanwhile, when Notch signaling was inhibited with DAPT, Notch1 and KDR expression declined significantly as compared with the untreated cells (both *P* < 0.05, Fig. 5B). Furthermore, DAPT attenuated the promoting effect of PDGF‐BB on endothelial differentiation of hEPCs/hMSCs cocultured cells (all *P* < 0.05). These findings indicated that both Notch and PDGF signaling pathways participated and crosstalked with each other in the angiogenesis‐promoting effect of hEPCs/hMSCs coculture.

### Coculture of hEPCs enhanced the proliferation of hMSCs

**Figure 6:**
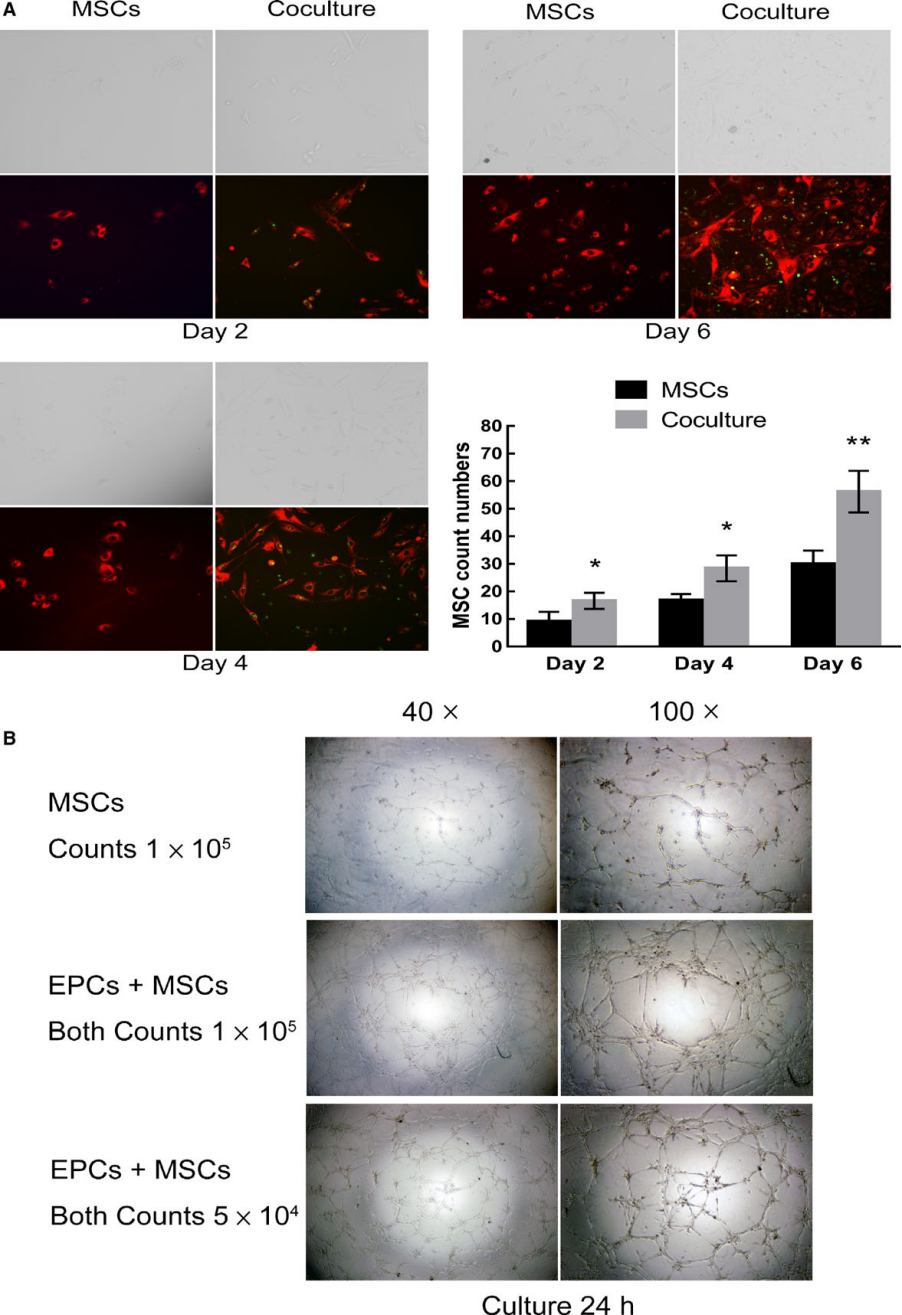
The effect of hEPCs coculture on the proliferation of hMCSs. (A) A coculture of DiD‐stained hMCSs (6 × 10^3^ cells) and DiO‐stained hEPCs (6 × 10^3^ cells) (coculture group) and a hMCS‐only group (6 × 10^3^ of DiD‐stained hMCSs) were seeded onto a six‐well plate. At Day 2, Day 4, and Day 6, the cells were observed under a fluorescence microscope (three wells for each group). For each well, five fields (at 100×) were randomly chosen and photographed, followed by quantification of cell fluorescence using image j software (NIH, USA). (B) Comparisons of *in vitro* angiogenic capacity between hEPCs/hMSCs coculture (10^5^ or 10^4^ of each cell type) and hMSC‐only (10^5^ cells) groups. *P < 0.05; **P < 0.01, compared to MSCs group.

To accurately evaluate the effect of hEPCs coculture on the proliferation of hMCSs, DiD‐stained hMCSs and DiO‐stained hEPCs were used in the proliferation assay. The results showed that coculture of hEPCs significantly enhanced the proliferation of hMSCs at Day 2, Day 4, and Day 6 as compared with hMCS‐only control (all *P* < 0.05, Fig. 6A).

As for angiogenic capacity, hEPCs/hMSCs coculture (10^5^ of each cell type) group can form better capillary networks as compared with the hMSC‐only (10^5^ cells) group at 24 h (Fig. 6B). Moreover, although the cell numbers were halved, hEPCs/hMSCs coculture (10^4^ of each cell type) group remained to form better capillary networks than the hMSC‐only (10^5^ cells) group, suggesting that coculture with hEPCs could improve the formation of capillary networks in the hEPCs/hMSCs coculture.

## Discussion

In the current study, we investigated the effects of hEPCs/hMSCs direct contact coculture on their proliferation and angiogenic capacities and their underlying mechanism. The results showed that cell proliferation was significantly improved by hEPCs/hMSCs 3D‐coculture and PDGF‐BB treatment, but significantly inhibited by AB‐PDGFR. The expressions of cyclin D1, PDGFR, Notch1, and Hes1 were significantly enhanced by PDGF‐BB but inhibited by DAPT. *In vitro* angiogenesis assay showed that hEPCs/hMSCs coculture and PDGF‐BB significantly enhanced angiogenic capacity, whereas AB‐PDGFR significantly reduced the angiogenic capacity in all groups. PDGF‐BB increased endothelial marker KDR expression and activated Notch1 signaling in cocultured cells, while DAPT attenuated the promoting effect of PDGF‐BB on endothelial differentiation of hEPCs/hMSCs coculture. Taken together, these findings suggested that hEPCs/hMSCs coculture enhanced their proliferation and angiogenic capacities and both PDGF and Notch signaling pathways participated and crosstalked with each other in these promoting effects. To our best knowledge, this is the first study reporting the roles of PDGF and Notch signaling pathways in promoting effects of hEPCs/hMSCs coculture on their biological functions.

Our proliferation data showed that hEPCs/hMSCs coculture significantly improved proliferation in 3D transwell chamber but not in 2D mixed monolayer coculture system, which is line with previous studies 30, 31. One possible explanation may be that in the 2D‐coculture, the direct contact between hMSCs and hEPCs induced a contact inhibition. However in the 3D‐coculture, the contact inhibition was prevented due to the complete separation between hEPCs and hMSCs by the membrane in transwell chamber 30. In addition, the membrane pores allowed a variety of hEPCs/hMSCs secreting growth‐promoting factors to pass through the membrane, leading to enhanced proliferation. MSCs represent a promising cell source for angiogenic therapies due to their capacity to differentiate into ECs and to form capillary networks 32, 33. Consistent with previous reports 15, 16, our *in vitro* angiogenesis assay showed that coculture of hEPCs and hMSCs markedly increased the thickness of capillary‐like structure and junction area at all three time points as compared with hMSC‐alone group. Particularly, at 48 h, when cell death was observed in both hEPCs and hMSC‐alone groups, cocultured cells remained to maintain good tubule morphology. These observations suggest that hEPCs/hMSCs coculture not only promoted the tubule formation but also prolonged cell survival, which can further improve the angiogenic potential.

Our data showed that PDGF‐BB significantly enhanced cell proliferation through upregulation of PDGFR‐β and cyclin D1. In addition, PDGF‐BB played a synergistic role in hEPCs/hMSCs coculture to further enhance proliferation, which is in agreement with other reports 34, 35. Meanwhile, when PDGFR‐β was blocked, cell proliferation, as well as the levels of PDGFR‐β and cyclin D1, declined coincidently, demonstrating that PDGF signaling was involved in the proliferation. The previous study has reported the enhancing effect of PDGF‐BB on vascularization 36. Our *in vitro* angiogenesis data showed that PDGF‐BB and hEPCs/hMSCs coculture also exhibited a synergistic effect on the angiogenic capacity. The promoting effects of hEPCs/hMSCs coculture on proliferation and angiogenesis may be attributed to the fact that both EPCs and MSCs secrete substantial amounts of PDGF‐BB 37, 38. Notch signaling has been reported to participate in the proliferation and angiogenesis 28. Our data showed that direct contact coculture markedly increased Notch1 expression, and DAPT inhibited expression of cyclin D1. Meanwhile, the expression of Notch1 significantly increased in endothelial differentiation of cocultured cells. DAPT decreased the level of KDR in endothelial differentiation of cocultured cells, indicating suppression of cell endothelial differentiation. These data supported that Notch signaling pathway was implicated in the promoting effects of hEPCs/hMSCs coculture on the proliferation and angiogenesis.

The association between PDGF and Notch signaling pathways has been reported previously. PDGFR‐β has been shown to be a target of Notch signaling gene in vascular smooth muscle cells 20. It has been reported that Notch1 signaling is an upstream of PDGF‐B transcription in human brain microvascular endothelial cells 39. On the other hand, PDGF‐BB can affect Notch1 activation and Notch1–Furin interaction 40. These findings suggest that crosstalk between PDGF and Notch signaling pathways is common in cell biological regulations. The current study showed that when PDGF signaling was activated in hEPCs/hMSCs coculture, Notch1/Hes1 levels also elevated simultaneously, resulting in an improvement of cell proliferation and endothelial differentiation. Coincidentally, when PDGF signaling was blocked, Notch1/Hes1 levels were also reduced, leading to inhibited cell growth and angiogenesis. On the other hand, DAPT attenuated the promoting effect of PDGF‐BB on endothelial differentiation of hEPCs/hMSCs cocultured cells. Taken together, these observations indicated that there was a crosstalk between PDGF and Notch signaling pathways in the cell proliferation and endothelial differentiation of cocultured hEPCs/hMSCs.

There are still some limitations in this study. First, in the coculture system, we cannot distinguish the individual contribution of hMSCs/hEPCs both in the western blot and in the angiogenic assays because we cannot conduct cell separation before assays. However, we can partially evaluate the individual contribution of hMSCs/hEPCs by comparing the western blot/angiogenic assay data between the coculture group and the hMSCs or hEPCs single culture group. In addition, we did not use Notch signaling activators to comprehensively evaluate the role of Notch signaling in hEPCs/hMSCs coculture. The angiogenic potential was assessed only in the 2D‐coculture, but not in the 3D‐coculture system. Furthermore, the *in vitro* findings of this study remain to be further validated using an *in vivo* model. All these limitations should be addressed in the following study.

In summary, our results showed that hEPCs/hMSCs coculture demonstrated significant enhancement effects on cell proliferation and angiogenic capacity in direct contact coculture. These promoting effects were involved in the crosstalk between PDGF and Notch signaling pathways. Our findings may be useful for the development of future applications in tissue engineering and therapeutic strategy.

## Materials and methods

### Isolation and culture of hEPCs and hMSCs

Bone marrow was collected from the drill holes of pedicle during the operations of patients with spine internal fixation (age range 22–56 years; mean age 43 years) with lumbar degenerative diseases (degenerative lumbar spondylolisthesis and lumbar spinal stenosis with instability). Informed consent was obtained from the patients for bone marrow collection, and all the procedures were performed in accordance with the guidance and approval of a research ethics committee in the First Affiliated Hospital of Sun Yat‐sen University.

Mononuclear cells were collected by Ficoll density gradient centrifugation (1.077; GE Health, Fairfield City, CT, USA) from the bone marrow at 300 ***g*** for 25 min. The nucleated cells were collected from the defined layer at the interface, diluted with two volumes of PBS, centrifuged twice at 100 ***g*** for 5 min, and finally resuspended in basal medium. For the isolation of hEPCs, the collected cells were cultured in dishes coated with fibronectin and induced by EGM‐2 MV Single‐Quots (Cambrex, East Rutherford, NJ, USA) at 37 °C with 5% CO_2_ in humidified air at a density of 5 × 10^5^ cm^−2^. After three days, nonadherent cells were washed out with PBS and cultured to Day 14. At Day 7 and Day 14, immunofluorescence staining and flow cytometry were applied to identify hEPCs. Quantitative fluorescence‐activated cell sorting (FACS) was performed on a FACS Vantage SE flow cytometer (Becton Dickinson, Lake Franklin, NJ, USA). The ability of tube formation by hEPCs was determined by *in vitro* angiogenesis assay.

For the isolation of hMSCs, the collected mononuclear cells were resuspended in basal medium at a density of 2 × 10^5^ cm^−2^ and maintained in a humidified atmosphere of 95% air and 5% CO_2_ at 37 °C. Basal medium consisted of Dulbecco's modified Eagle's medium with Glutamix‐1, sodium pyruvate, 4500 mg·L^−1^ glucose, and pyridoxine (DMEM; Gibco, BRL, Gaithersburg, MD, USA) supplemented with 10% inactivated fetal bovine serum (FBS; Gibco, BRL) and 1% penicillin/streptomycin (Sigma, St. Louis, MO, USA). After three days, the medium was changed to remove all nonadherent cells. Thereafter, the medium was changed twice a week until subconfluence. When 80% confluent, the cells were detached using 0.125% trypsin/5 mm ethylenediaminetetraacetic acid (Sigma), then placed in the basal medium and expanded with 1 : 3 ratio. The third passage of hMSCs was used in all the experiments. Flow cytometry was used to identify hMSC phenotypes at all samples. The capacity of multilineage differentiation of hMSCs, including osteogenesis, chondrogenesis, and adipogenesis, was detected for further identification at Day 21. The first‐passage (P1) hEPCs and the third‐passage (P3) hMSCs were used in all the following experiments.

### Double staining for hEPCs

Passage 1 hEPCs were incubated with 2.5 μg·mL^−1^ of 1, 1′‐dioctadecyl 3,3,3′,3′‐tetra‐methylindo‐carbocyanine‐labeled acetylated low‐density lipoprotein (DiI‐acLDL; Invitrogen, Carlsbad, CA, USA) and 10 μg·mL^−1^ of fluorescein isothiocyanate (FITC)‐conjugated factor VIII, ulex europaeus agglutinin‐1 (Sigma) for 3 h at 37 °C, and the cells were examined under a fluorescence microscope (Nikon, Tokyo, Japan).

### Flow cytometric analysis

hEPCs were characterized for immunophenotype using monoclonal antibodies (MoAbs) specific for CD133, CD34, KDR, vWF, E‐selectin, and VE‐cadherin at Days 7 and 14. hMSCs were incubated specifically with CD105, CD73, CD29, CD44, CD45, CD14, HLA‐DR, and CD90 antibodies at the third passage. All antibodies were purchased from Pharmingen/Becton Dickinson (PharMingen, San Diego, CA, USA). Cells were detached using trypsin/EDTA for 5 min, immediately washed with PBS to remove trypsin, and resuspended at 10^6^ mL^−1^. Cell suspension (100 mL) was incubated at 4 °C for 10 min with 15% FBS, followed by incubation with the specific antibody at 4 °C for 30 min. Cells were washed with PBS. At least 10 000 events were analyzed by flow cytometry (FACScali‐bur; Becton Dickinson, Milan, Italy) using cell quest software.

### Osteogenic, adipogenic, and chondrogenic differentiations of hMSCs

For osteogenic differentiation, hMSCs at the third passage were seeded at a concentration of 0.8 × 10^4^ cm^−2^ in a six‐well plate. When confluent, the cells were detached using 0.125% trypsin/5 mm EDTA and then placed in the basal medium. After 1‐day culture, the medium was replaced with osteogenic medium consisting of basal medium supplemented with 10 nm dexamethasone, 10 mm β‐glycerol‐phosphate, and 0.2 mm ascorbic acid (all from Sigma). At Day 21, cells were fixed with ice‐cold 70% ethanol for 30 min, washed with PBS for three times, and stained with Alizarin Red S (40 mm, PH 4.2; Sigma) for 30 min, and rinsed with PBS for three times. The dish area was observed with a light microscope.

For adipogenic differentiation, cells were seeded at a concentration of 2.5 × 10^4^ cm^−2^ in a six‐well plate. Adipogenic differentiation was induced with adipogenic medium, containing DMEM, 10% FBS, 10^−6^ m dexamethasone, 0.2 mm indomethacin, 10 μg·mL^−1^ insulin, and 100 ng·mL^−1^ 3‐isobutyl‐L‐methylxanthine (all from Sigma Immunochemicals, Sigma‐Aldrich Co., St. Louis, MO, USA). At Day 21, cells were examined for the presence of lipid vacuoles using Oil Red O staining. Briefly, cells were fixed in 10% formaldehyde in phosphate buffer for 1 h, washed with 60% propylene glycol for 3 min, stained with 0.18% Oil Red O (Sigma) for 10 min, rinsed with water, and counterstained with hematoxylin for 10 min.

For chondrogenic differentiation, hMSCs (2.5 × 10^5^) were centrifuged in a 15‐mL polypropylene Falcon tube to form a pellet. Chondrogenic medium consists of DMEM, 10% FBS, 37.5 mg·mL^−1^ ascorbic acid, 10 nm dexamethasone, 1 : 100 ITS premix (BD Biosciences, San Jose, CA, USA), and 10 ng·mL^−1^ human recombinant TGF‐β3 (R&D Systems, Minneapolis, MN, USA). The medium was changed twice a week. Presence of proteoglycan (PG) was analyzed by means of 0.1% toluidine blue staining (Sigma).

### Determining the proliferation in hEPC/hMSC coculture

Cell proliferation was determined by 2‐(2‐methoxy‐4‐nitrophenyl)‐3‐(4‐nitrophenyl)‐5‐(2,4‐disulfo‐phenyl)‐2H‐tetrazolium, monosodium salt (WST‐8) assay kit (CCK‐8; Dojindo, Mashikimachi, Japan). Briefly, WST‐8 was added to each well for 4 h before the measurement. The absorbance at 450 nm was measured using a microplate reader (Thermo Fisher Scientific, Waltham, MA, USA). For PDGF‐BB (PeproTech, Rocky Hill, NJ, USA) treatment, cells were cultured in 2% FBS DMEM medium containing 2 ng·mL^−1^ PDGF‐BB for 6 days. To block the PDGF receptors, 20 μg·mL^−1^ of neutralization antibody against PDGFR‐β (R&D Systems) was used in corresponding groups. To inhibit Notch signaling, 50 μmol·L^−1^ DAPT (γ‐secretase inhibitor, dissolved in dimethyl sulfoxide, purchased from Sigma) was used.

**Figure 7:**
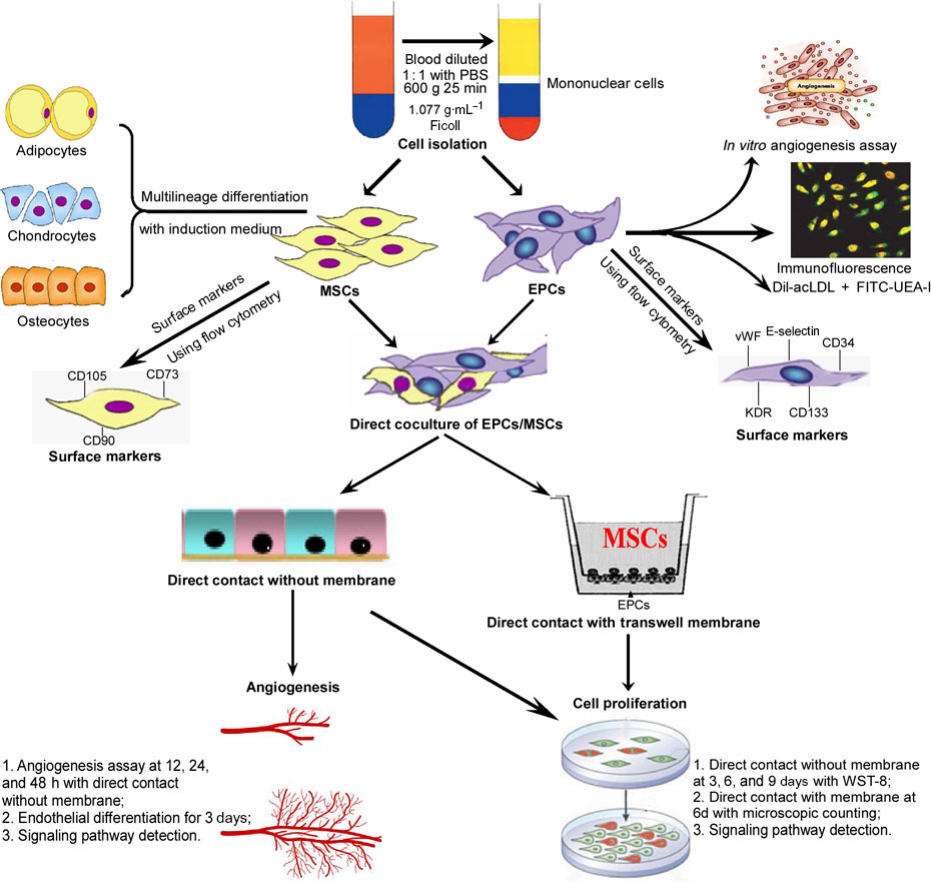
A schematic flow diagram of this study. hEPCs and hMSCs were isolated from human bone marrow. The isolated hEPCs were characterized with *in vitro* angiogenic potential and the specific markers. The isolated hMSCs were characterized with the multilineage differential potential and the specific surface markers. Then hEPCs and hMSCs were cocultured in a two‐dimensional (2D) monolayer mixed or 3D transwell membrane cell‐to‐cell coculture systems. The proliferation and angiogenic capacities of cells were assessed, and the underlying mechanism was investigated.

Two kinds of coculture methods were utilized in this assay (Fig. 7). The first coculture system was a traditional mixed monolayer (2D) system. Briefly, hEPCs and hMSCs were mixed (1 : 1 ratio) in DMEM supplemented with 10% FBS and 1% penicillin/streptomycin (Invitrogen) and seeded in 96 wells. At Days 3, 6, and 9, CCK‐8 was used for cell proliferation detection according to the manufacturer's instructions. The second coculture system was a three‐dimensional (3D) cell‐to‐cell method using a six‐well culture plate and inserts (Corning, Sullivan Park, NY, USA), containing a polyethylene terephthalate track‐etched membrane with 0.4‐μm pores at the bottom of inserts. hMSCs were seeded onto the membrane of each culture insert that has 4.2 cm^2^ of available culture area at 1 × 10^4^ cells. hEPCs were cocultured with hMSCs through direct cell‐to‐cell contact in monolayer on the opposite side of the membrane, the bottom of the culture insert. After culture for 6 days, the average number of cells in ten random microscopic 200× fields was determined manually.

To accurately evaluate the effect of hEPCs coculture on the proliferation of hMCSs, hMCSs and hEPCs were stained with DiD and DiO fluorescent dyes (Thermo Fisher Scientific) for membrane labeling, according to the manufacturer's protocol, and were used in the proliferation assay. A coculture of DiD‐stained hMCSs (6 × 10^3^ cells) and DiO‐stained hEPCs (6 × 10^3^ cells) (coculture group) was seeded onto a six‐well plate. Meanwhile, a hMCS‐only group (6 × 10^3^ of DiD‐stained hMCSs) was also seeded onto a six‐well plate and incubated in 37 °C incubator. At Day 2, Day 4, and Day 6, the cells were observed under a fluorescence microscope (three wells for each group). For each well, five fields (at 100×) were randomly chosen and photographed, followed by quantification of cell fluorescence using image j software (NIH, USA).

### Western blot analysis

In order to further investigate cell proliferation, cocultures of hMSCs and hEPCs were harvested and total proteins were extracted for western blot analysis at Day 6. hEPCs, hMSCs, and coculture cells were collected and lysed with ice‐cold lysis buffer (Merck, Novagen, Temecula, CA, USA). Protein concentrations were determined with the Bio‐Rad assay system (Bio‐Rad, Hercules, CA, USA). Thirty micrograms of total proteins was separated on a 10% SDS/polyacrylamide gel. Bands on the gels were transferred onto polyvinylidene fluoride membranes (Millipore Corp, Billerica, MA, USA). The membranes were blocked for 1 h with 5% nonfat milk or BSA in PBS with 0.1% Tween 20. Blots were incubated with primary antibody overnight at 4 °C followed by secondary antibodies for 1 h each at room temperature. Immunoreactive bands were visualized with Chemiluminescence Reagent Plus (Thermo Fisher Scientific) and exposed to X‐ray film (Fujifilm, JP). The membranes were then incubated with stripping buffer (Pierce, Holmdel, NJ, USA) for 30 min at 37 °C, reblocked, and reprobed with β‐actin as a loading control. The proteins were detected with specific cyclin D1, Notch1 intracellular domain (N1ICD, cat. no. ab83232), Hes1, KDR (1 : 1000, all above from Abcam, Cambridge, UK) and PDGF receptor β antibodies (Ab‐PDGFR, 1 : 1000, from Cell signaling Technology, Danvers, MA, USA). Western blot bands were quantitated using quantity one software (Bio‐Rad, USA).

### *In vitro* angiogenesis assay for hEPC/hMSC coculture

Angiogenesis assay for capillary‐like tube formation was performed with an *In Vitro* Angiogenesis Assay Kit (Chemicon, Temecula, CA, USA) according to the manufacturer's instructions. Briefly, ECMatrix solution was thawed on ice overnight, mixed with 10× ECMatrix™ diluents, and placed in a 96‐well tissue culture plate at 37 °C for 1 h to allow the matrix solution to solidify. After starved for 12 h, 1 × 10^4^ cells containing hEPCs and hMSCs mixed with a 1 : 1 ratio were seeded on the top of the solidified matrix solution in each 24‐well with 2% FBS DMEM. At 12, 24, and 48 h, tubule formation was inspected under an inverted light microscope at 100× magnification. Tubule formation was defined as a structure exhibiting a length four times its width. Five independent fields were assessed for each well, and the average number of tubules/100× fields was determined.

For endothelial differentiation in coculture, hEPCs and hMSCs were mixed (1 : 1 ratio) in EC growth medium (EGM‐2) supplemented with 2% FBS and seeded in 1 μg·mL^−1^ fibronectin‐coated wells. For control conditions, non‐cocultured cells in the same medium were used. After 3‐day endothelial induction, hMSCs and hEPCs coculture were harvested and total proteins were extracted for western blot analysis.

### Statistical analysis

All values are presented as mean ± standard deviation of the mean. Data were analyzed using the Student's *t*‐test or a general linear model two‐way ANOVA with a *post hoc* Tukey test to compare between groups as appropriate. The value of *P* < 0.05 was considered statistically significant. All analyses were performed using ibm spss version 17 (IBM Corporation, Somers, NY, USA).

## Author contributions

We declare that all the listed authors have participated actively in the study and all meet the requirements of the authorship. DS and TL designed the study and wrote the protocol. TL, LZ, and WG performed research/study. LZ contributed important reagents. MG and JR managed the literature searches and analyses. HY and KW undertook the statistical analysis. DS and TL wrote the first draft of the manuscript.
